# Mortality and life expectancy in Kiribati based on analysis of reported deaths

**DOI:** 10.1186/s12963-016-0072-6

**Published:** 2016-02-29

**Authors:** Karen L. Carter, Tibwataake Baiteke, Tiensi Teea, Teanibuaka Tabunga, Mantarae Itienang, Chalapati Rao, Alan D. Lopez, Richard Taylor

**Affiliations:** School of Population Health, Faculty of Health Sciences, University of Queensland, St Lucia, Brisbane, Queensland 4072 Australia; Kiribati Ministry of Health (MoH), Tarawa, Kiribati; Kiribati Civil Registration Office (CRO), Ministry of Women, Youth and Communities, Tarawa, Kiribati; Secretariat of the Pacific Community (SPC), B.P. D5 Noumea Cedex, 98848 New Caledonia; Global Health Division, Research School of Population Health, Australian National University (ANU), Acton, Canberra, Australian Capital Territory (ACT), 2601 Australia; Melbourne School of Population and Global Health, University of Melbourne, Parkville, Melbourne, Victoria, 3010 Australia; School of Public Health and Community Medicine (SPHCM), University of New South Wales (UNSW), Randwick, Sydney, New South Wales 2052 Australia

**Keywords:** Kiribati, Mortality, Deaths, Under-5 mortality, Adult mortality, Life expectancy

## Abstract

**Background:**

Kiribati is an atoll country of 103,058 (2010 Census) situated in the central Pacific. Previous mortality estimates have been derived from demographic analyses of census data. This is the first mortality analysis based on reported deaths.

**Methods:**

Recorded deaths were from the Ministry of Health and the Civil Registration Office for 2000–2009; populations were from the 2000, 2005, and 2010 censuses. Duplicate death records were removed by matching deaths within and between data sources using a combination of names, date of death, age, sex, island of residence, and cause of death. Probability of dying <5 years (_5_q_0_) and 15–59 years (_45_q_15_), and life expectancy (LE) at birth, were computed with 95 % confidence intervals. These data were compared with previous census analyses.

**Results:**

There were 8,681 unique deaths reported over the decade 2000–2009 in Kiribati. The reconciled mortality data indicate _5_q_0_ for both sexes of 64 per 1,000 live births in 2000–2004, and 51 for 2005–2009 (assuming no under-enumeration), compared with 69 and 59 for comparable periods from the 2005 and 2010 census analyses (children ever-born/children surviving method). Based on reconciled deaths, LE at birth (e_0_) for males was 54 years for 2000–2004 and 55 years in 2005–2009, five years lower than the 2005 and 2010 census estimates for comparable periods of 59 and 58 years. Female LE was 62 years for 2000–2004 and 63 years for 2005–2009, two-three years less than estimates for comparable periods of 63 and 66 years from the 2005 and 2010 census analyses. Adult mortality (_45_q_15_) was 47-48 % in males and 27-28 % in females from reconciled mortality over 2000–2009, higher than census estimates of 34-38 % in males and 21-26 % in females for the same periods. The reconciled data are very likely to be incomplete and actual mortality higher and life expectancy lower than reported here.

**Conclusion:**

This analysis indicates higher mortality than indirect demographic methods from the 2005 and 2010 Censuses. Reported deaths are most likely under-reported; especially _5_q_0_, as many early neonatal deaths are probably classified as stillbirths. These analyses suggest that the health situation in Kiribati is more serious and urgent than previously appreciated.

**Electronic supplementary material:**

The online version of this article (doi:10.1186/s12963-016-0072-6) contains supplementary material, which is available to authorized users.

## Introduction

Kiribati is a group of coral atoll islands (average height above sea level of 0.6 m) spread across over 4 million square kilometers of the Pacific Ocean, spanning both the equator and the International Date Line. Kiribati became independent in 1979, having previously been a British colony. The population was 103,058 residents (2010) of which 49 % are concentrated on the South Tarawa atoll [1], which has one of the highest population densities in the world [2]. As an atoll country, Kiribati has limited agricultural potential and is classified amongst the least developed countries with an estimated gross domestic product (GDP) per capita of $1,500 in 2010 [3].

Kiribati has been affected by both undernutrition, including vitamin A deficiency, especially in children in outer islands [4], and non-communicable disease (NCD) in adults, particularly in urban South Tarawa [5–7]. As a consequence of under-enumeration of deaths in vital registration systems, estimates for Kiribati of child and adult mortality, and life expectancy, have been produced based on demographic analyses of censuses and surveys. Such methods employ direct questions concerning deaths in retrospective periods, and/or indirect demographic techniques such as questions for women relating to numbers of children ever-born and children surviving (CEBCS) [8, 9], and questions of adults concerning vital status of parents (orphanhood method) or (first) spouse (widowhood method) [8–11]. From such questions estimates are calculated for <5 years and adult mortality. Data from these methods are then often combined with model life tables judged to be appropriate to produce age-specific mortality and life expectancy [12, 13]. In Pacific countries it has been noted that imputation using model life tables, especially from child mortality data alone, often results in spuriously low adult mortality and implausibly high life expectancy; this can lead to erroneous conclusions concerning the level of premature adult mortality from NCD – such as has been demonstrated in Fiji [14] and Tonga [15, 16].

There are two sources of routinely reported mortality data available in Kiribati: one from civil registration and the other derived from the Health Information System (HIS). These sources are largely independent with no direct data sharing and limited provisions to require a medical certificate prior to registering a death [17]. Little is known of the completeness of each of these systems. A more reliable estimate of age-specific mortality in Kiribati based on reconciliation of deaths from these two sources is thus both feasible and likely to be informative for guiding policy responses to reduce premature mortality.

## Methods

### Data sources

Recorded deaths were sourced from the death registration systems of the Kiribati Ministry of Health (MoH) and the Kiribati Civil Registration Office (CRO) for the years 2000–2009. There were 8,728 unique deaths reported (through both sources) over this period in Kiribati, an average of 873 deaths per year (Table 1). The CRO captured 59 % of these deaths while 63 % were recorded by the MoH. There is no significant variation in the number of reported deaths by year (data not shown). Deaths in I-Kiribati resident overseas (n = 22) recorded in the Civil Registration data were excluded since the denominator for mortality was residents. A further 20 deaths from CRO and four from MoH data were excluded as there were no names recorded. A significant number of duplicate registrations were removed. In discussion with both MoH and CRO staff, it was evident that early neonatal deaths are frequently recorded as stillbirths in both reporting systems. Populations for each time period 2000–2004 and 2005–2009 were derived from the 2000 [18], 2005 [19] and 2010 [20] censuses, with interpolation between censuses using an exponential growth rate (see Additional file 1).

**Table 1:** Reported deaths by source, Kiribati, 2000-2009

Age group years	2000-2004					2005-2009					2000-2009		
	Reported deaths				Unique deaths	Reported deaths				Unique deaths	Reported dth		Unique deaths
	CRO		MoH			CRO		MoH			CRO	MoH	
	n	%	n	%	n	n	%	n	%	n	%	%	n
<5	212	26.3	682	84.6	806	205	27.9	635	86.5	734	27.1	85.5	1540
5-14	103	54.4	129	68.6	188	72	56	90	69.3	129	55.2	69.0	317
15-24	182	68.5	141	53.6	263	166	56.6	177	60.4	293	62.6	57.2	555
25-34	230	66.5	190	55.6	342	216	62.1	208	59.8	348	64.7	57.7	689
35-44	396	76.7	264	51.6	511	352	66.8	326	61.8	527	72.1	56.8	1038
45-54	413	72.1	309	54.6	566	496	67.9	462	63.3	729	70.2	59.5	1296
55-64	387	68.7	315	56.6	557	341	60.5	380	67.4	564	65.0	62.0	1120
65-74	330	63.5	322	62.6	514	404	61.1	411	62.2	661	62.5	62.4	1175
≥75	308	68.3	227	50.8	447	334	66.5	268	53.2	503	67.6	52.1	950
Total	2561	60.4	2579	61.5	4194	2586	57.6	2956	65.8	4487	59.3	63.8	8681
Unkn^+^	85		125		157	228		481		562			719

^+^Unknown (Unkn) deaths redistributed into age groups according to proportion of deaths by known age

*CRO* Civil Registry Office, *MoH* Ministry of Health

%: proportion of unique deaths reconciled between the CRO and MoH death data

**Table 2:** Kiribati mortality and life expectancy estimates, 2000-2009

Data source and period	IMR (_o_/^oo^)	Child <5 years mortality _o_/^oo^	Adult mortality 15–59 years (_o_/^o^) (95 % CIs)		Life expectancy at birth (years) (95 % CIs)	
	Both sexes (95 % CIs)		Male	Female	Male	Female
Census estimates						
2005 census ^a^	52	69	34.1	26.3	58.9	63.1
2010 census ^b^	45	59	38.3	21.1	58.0	66.3
Reconciled mortality data from MoH and CRO						
2000-2004	47.2	63.8. (59.4-68.1)	47.9 (44.8-51.0)	28.4 (26.0-30.7)	53.9 (53.3-54.6)	61.7 (61.0-62.5)
2005-2009	38.7	51.0 (47.1-54.8)	47.0 (44.2-49.9)	27.3 (25.2-29.5)	54.8 (54.2-55.5)	63.0 (62.3-63.6)

*MoH* Ministry of Health, *CRO* Civil Registry Office

Childhood mortality: probability of dying from ages 0–4 years from <5 deaths and population using life table _5_q_0_ (per 1000). Infant mortality rate (IMR): deaths per 1000 live births (_1_q_0_); estimated from a regression analysis of international <5 mortality and IMR data from 1990, 2000 and 2010

Adult mortality: probability (%) of dying from ages 15–59 years (_45_q_15_)

^a^Based on children ever-born and children surviving (CEBCS) for <5 mortality with an imputed Far East Asian model life table

^b^Based on CEBCS for <5 mortality and adult mortality from the parental orphanhood method with and imputed Far East Asian model life table

95 % confidence intervals (CIs) for reconciled mortality rates from variance of probability of dying (_n_q_x_) using Chiang life table method

Life tables are in the Additional file 1

### Reconciliation of deaths

Criteria for matching records within and between the two sources were based on variables available, developed in conjunction with the CRO and MoH staff, and trialled on the collected data. A set of criteria was developed to minimize the potential for over-matching (falsely matching records together that do not refer to the same individual), thus underestimating deaths, and equally minimize potential for under-matching leading to overestimation of deaths. The final criteria selected for records to be considered a “match” were: (1) surname (minor spelling variations allowed), plus two of either: first name (minor spelling variations allowed, or English versions of the same name), island (place of death), or date of death (within five days); or (2) first name, island, date of death, age and gender; or (3) either surname or first name, island, age, gender, and (specific) cause – all within same month of death. All matching was carried out manually, by sorting the lists by each of the matching criteria successively to identify potential matches for review. Where data on an individual differed, the information in the CRO was preferred because it emanated directly from families. MoH data were first reconciled internally across the HIS death registry, hospital records, and nurses’ reports before reconciliation with CRO. Duplicate records from each of the CRO and MoH data lists were first identified and removed, and then duplicates from the combined CRO and MoH lists were removed to produce the reconciled death data set for each year for the periods 2000–2009.

This reconciled data set of deaths by age group and sex included deaths of unknown age (8.3 % of the total) which were redistributed into age groups according to the proportional age distribution of deaths by age and by sex (Table 1). Life tables were produced for male and female I-Kiribati for 2000–2004 and 2005–2009 based on age-specific mortality rates.

### Mortality and life expectancy

Mortality rates under age 5 years (U5M) are calculated as the probability of dying at ages 0–4 years (_5_q_0_) based on the death rate (_5_m_0_) <5 years using the life table method and variance estimation by Chiang [21]. The infant mortality rate (IMR) is imputed from U5M (_5_q_0_) using a quadratic regression equation (R2 = 0.98) based on international data [22] from 1990, 2000, and 2010 for all countries with both measures published contemporaneously. Adult mortality for 15–59 years is calculated as the probability of dying (_45_q_15_) between the ages of 15 and 59 years, derived from adult age-specific death rates using life table methods [11], and cumulative risk calculations (with 95 % CIs) [23], which produced similar results. Life expectancy at birth is derived from life table calculations [11, 24]. The terminal Lx for the open-ended age group (≥75 years) is calculated according to the Pollard approximation as l_x_*log_10_(l_x_) [24]. Confidence intervals (95 %) for life expectancies are calculated according to the Chiang method [21] using age-specific mortality.

### Comparisons

Findings based on reconciled recorded deaths were compared with census estimates for the same period. Estimates of infant and child mortality from Ministry of Health data [25] are not adjusted for under-enumeration and have not been included in comparisons.

All Kiribati mortality estimates have been based on demographic analyses of previous censuses over the period 1973–2010 [19, 20] generated from <5 years mortality from CEBCS [8, 9] alone, or combined with adult survivorship data derived from the orphanhood method [8–11], with imputation of model life tables from these data based on one or both parameters [26–29]. Analyses of the 2005 census used only <5 years mortality from CEBCS to impute model life tables (UN Far East Asian pattern) (one-parameter method) [19], while the 2010 census analysis used <5 mortality and adult survivorship (parental orphanhood method) to impute model life tables (UN Far East Asian pattern) (two-parameter method) [20]. Life tables are not available from the 2000 census.

## Results

### Reconciled deaths

Deaths registered through the CRO were 59 % of total unique deaths (2000–2009), and deaths recorded by the MoH were 64 %. Under-5 mortality (both sexes combined) decreased from 63.8 deaths per 1,000 live births in 2000–2004 to 51.0 in 2005–2009 (Table 2). Adult mortality at ages 15–59 years (_45_q_15_) was estimated as 47.9 % for males and 28.4 % for females in 2000–2004, and slightly lower in 2005–2005 of 47 % for males and 27.3 % for females (Table 2). Life expectancies (at birth) were estimated to have been 53.9 years for males, and 61.7 years for females in 2000–2004, and one year higher in 2005–2009 at 54.8 years for males, and 63.0 years for females (Table 2). Life tables are in the Additional file 1.

### Secular changes

Based on reconciled deaths, over 2000–2004 to 2005–2009 child (U5M) mortality fell from 64 to 51 per 1000 births (statistically significant according to 95 % confidence intervals, and life expectancy rose from 53.9 to 54.8 years in men and 61.7 to 63.0 years in women (not statistically significant) (Table 2).

### Comparisons with census estimates

The estimate of U5M from reconciled deaths for 2000–2004 of 64 per 1,000 live births is slightly lower than the 2005 census estimate of 69, as is the 2005–2009 estimate of 51 per 1,000 compared with the 2010 census estimate of 59. From reconciled reported death data (assuming no under-enumeration of deaths), LE at birth is five years lower for males for 2000–2004 and three years lower for 2005–2009; for females LE was 1.5 years lower for 2000–2004, and by 3.3 years compared to the 2005 and 2010 census estimates.

## Discussion

The reconciled mortality data based on reported deaths indicate that LE for 2000–2004 was 54 years in 2000–2004 and 55 in 2005–2009 for males, while LE for females was 62 and 63 years for the same periods. These estimates are lower than LE reported from census analyses employing indirect demographic methods for males of 58 years in 2000–2004 and 59 years in 2005–2009, and LE for females of 63 and 66 over the same period. Significant under-enumeration of deaths is also likely in view of the reporting mechanisms operating in Kiribati, particularly as there is little incentive for registration of deaths and burial occurs at home.

IMR derived from U5M for 2000–2004 was estimated at 47 deaths per 1,000 live births, and 39 per 1,000 for 2005–2009, slightly less than 52 at the 2005 census and 45 at the 2010 census. However, knowledge of vital registration in Kiribati at the time suggests that the IMR would be higher due to under-enumeration. There was a statistically significant reduction in childhood mortality (<5 mortality) over 2000–2004 and 2005–2009 (based on reconciled deaths), from 64 deaths per 1,000 live births to 51 per1,000, although this decline may not be sufficient for Kiribati to meet its Millennium Development Goal (MDG 4) target which requires an U5M rate of 30 deaths per 1,000 live births by 2015 [30]. This is despite the significant attention given by the national government and international donors to improve public health and health services for children over this period.

The total estimated deaths by age group and sex per year from the 2005 and 2010 census analyses [19, 20] are similar to the total deaths identified from reconciled vital registration data. This would indicate that either nearly all deaths in Kiribati are reported to at least one of the routine reporting systems, which isunlikely, or that the census analysis has under-estimated mortality. The 2005 census analysis used U5M from census questions on number of CEBCS [8, 9] from female respondents, tabulated by age, to determine mortality in this age group. This method depends on accurate reporting of child mortality and also on accurate age-specific fertility data. For the 2005 census analysis, life tables by sex were imputed from U5M (one parameter method) to infer the full schedule of corresponding age-specific mortality rates by employing model life tables (East Asian pattern). The 2010 census analysis used under-5 mortality from CEBCS and estimates of adult mortality from the parental orphanhood method [8–10, 31] (two parameter method) to impute the full schedule of age-specific mortality using model life tables (East Asian pattern). As with the CEBCS method, other indirect methods including the orphanhood method rely on accurate age reporting of respondents to distribute the imputed deaths over time. Moreover, model life tables may also significantly under-estimate adult mortality in societies affected by a double burden of disease with considerable premature adult mortality from external causes, HIV/AIDS and/or non-communicable disease [32].

Reconciliation of deaths involved matching the CRO and MoH records to de-duplicate the data. The variation in names used to identify an individual in Kiribati increased the potential for deaths remaining unmatched if other variables were poorly recorded or absent. The variations in names used for identification vary for a variety of reasons including the interchangeable use of given name versus baptismal name as a first name, variations in spelling, and interchangeable I-Kiribati and English spellings. Adoption is also a common practice in Kiribati, and people may use either (or both) the name of their birth father or the name of their adoptive father. This is further complicated as it is usually the father’s first name that is used as a surname, but the CRO and some of the outer island health offices record “father’s name” of the deceased rather than surname, and either the first or last name of the father may be recorded in this field. Matching criteria were developed and trialled on a sample data set taken from the records, with multiple variations tested by staff from both the Civil Registry and MoH to ensure the least number of missed matches. The final criteria selected allow names to be excluded from the requirements for a match to account for the problems outlined earlier. The data set including matched and unmatched deaths was reviewed independently by both MoH and Civil Registry staff to minimize possible errors. Missed matches are more likely to have occurred amongst young children as this is the age group in which baptism and adoptions occur, and individuals are less likely to have established their own name.

Extensive discussions with a range of CRO and MoH staff produced a general consensus that early neonatal deaths (up to about 3 days old) are usually recorded as stillbirths (considered more socially acceptable to the family) in both the Civil Registry and MoH (community nursing) data, which is supported by observations on notifications of infant deaths in 1991–1994 mentioned in a 1995 UNICEF report [33]. Thus the estimates of U5M (and thus IMR) reported from reconciled data are likely to be under-enumerated. If all stillbirths were reclassified as neonatal deaths, then the U5M rates (_5_q_0_) for 2000–2009 would be approximately 10 % higher than reported here in the reconciled data.

LE in Kiribati is very unlikely to be higher than the estimates of 55 years for males and 63 years for females (2005–2009) based on reconciled deaths. These estimates indicate that LE in Kiribati is lower than previously reported. World Health Organization (WHO) estimates that Kiribati LE in 2009 was 65 years for males and 70 for females [34]; United Nations estimates for 2005–2010 are 62 years for males and 68 years for females [35]. Published estimates of LE are from SPC of 62 years (both sexes) derived from the 2010 Kiribati census analysis [36] of males 58 years and females 66 years – although these are three years higher (for each sex) than the reconciled deaths from this study of 55 years for males and 63 years for females (2005–2009). The Global Burden of Disease (GBD) 2010 estimates of 61 years (both sexes) [32], and 58 years for males and 67 years for females in 2013 [37], are similar to the estimates from the Kiribati 2010 census. Reconciled deaths from this study are likely to be under-registered, and thus LE estimates based on these data are probably too high.

Differences between estimates from the 2005 Kiribati Census [19], and the results of analyses based on reconciliation of deaths, may be partly a consequence of census methods utilizing CEBCS questions, followed by imputation of life tables (Far East Asian) models using U5M as a single input parameter [19]. Assessments from other Pacific island countries, including Fiji [14] and Tonga [15, 17], have demonstrated that these model life tables are not appropriate where premature adult mortality is considerable; especially for males, which is obvious in the reconciled Kiribati mortality data. The 2010 census analysis employed a two-parameter method utilising CEBCS and questions on spousal/parental survival to produce adult mortality [20] which was associated with higher census adult mortality in 2005–2009 in males, but a lower adult mortality for females. Nevertheless, adult mortality in 2005–2009 appears 10 % higher from reconciled mortality data for men, and 6 % higher for women, compared to 2010 census estimates.

Despite flaws in the reported mortality data, the findings presented here demonstrate the importance of using empirical death data to estimate mortality levels and patterns to ensure the most reliable evidence is being used to support national health priority setting. These results suggest that the health situation in Kiribati is worse than is thought based on findings from demographic analyses of census data, previously the only source of information on mortality. This analysis highlights the problems associated with relying on census estimates that impute adult mortality from model life tables where premature adult mortality may be considerable.

There is clearly a need to improve routine data collections in Kiribati through standardizing and rationalizing data collection responsibilities across government, paying much greater attention to reconciling data within and between sources, managing duplicates in reported data, and educating data collectors in standard definitions (such as for stillbirths). Kiribati has established a national Civil Registration and Vital Statistics (CRVS) committee and is in the process of developing a plan to address these issues with support from a range of partner agencies through the Pacific Vital Statistics Action Plan [38].

The reconciled death data indicate that the health situation in Kiribati needs urgent attention. The estimated IMR (2005–2009) of 38 and childhood mortality of 51 per 1,000 live births are comparatively high for Pacific island countries [36], although these figures would be even higher if those neonatal deaths classified as stillbirths and likely under-enumerated deaths were included were included. These estimates suggest some progress in reducing child mortality in the last decade, but Kiribati is unlikely to achieve MDG 4 (U5M). Premature adult mortality is considerable and life expectancy lower than many Pacific island countries, and is comparable to Nauru [39] with little improvement apparent over the past two decades [19, 20].

## Conclusions

A detailed examination of cause of death by age and sex would both shed further light on the contribution of particular causes to the high mortality and low life expectancies estimated for Kiribati and help to better elucidate the implications for targeted public health interventions.

## Consent

This study was based on routinely collected mortality data on deceased persons after death, and deaths are presented in aggregate only by source, period, age group and sex.

## Additional file

Additional file 1:
**PopLifeTabKir2000-09.** (DOCX 52 kb)

## Abbreviations

CEBCS: children ever-born and children surviving
CI: confidence interval
CRO: Kiribati Civil Registration Office
CRVS: Civil Registration and Vital Statistics
GBD: Global Burden of Disease
GDP: gross domestic product
HIS: Health Information System
IMR: infant mortality rate
LE: life expectancy
MDG: Millennium Development Goal
MoH: Kiribati Ministry of Health
NCD: non-communicable disease
SPC: Secretariat of the Pacific Community
SPHCM: School of Public Health and Community Medicine
U5M: under-5 mortality
UNSW: University of New South Wales
WHO: World Health Organization

## References

[1] Kiribati 2010 census. Analytical report. Kiribati National Statistics Office and the Secretariat for the Pacific Community (SPC) Statistics for Development Programme. SPC 2013. http://www.mfed.gov.ki/statistics/kiribati-document-library. Accessed 20 Feb 2016.

[2] Secretariat for the Pacific Community (SPC) (2007). SPC SaDP, Office KS.

[3] DFAT 2010. Kiribati country fact sheet (pdf). http://dfat.gov.au/geo/kiribati/pages/kiribati.aspx. Accessed 20 Feb 2016.

[4] Schaumberg DA, O’Connor J, Semba RD (1996). Risk Factors for Xerophthalmia in the Republic of Kiribati. Eur J Clin Nutr.

[5] King H, Taylor R, Zimmet P, Pargeter K, Raper R, Beriki T, et al. Non-insulin-dependent diabetes (NIDDM) in a newly independent Pacific nation: the Republic of Kiribati. Diabetes Care. 1984;VII(5):409–15. 10.2337/diacare.7.5.409 6499635

[6] Collins V, Taylor R, Zimmet P, Raper LR, Pargeter K, Geddes W, et al. Impaired glucose tolerance in Kiribati. N Z Med J. 1984;97(768):809–12. 6334253

[7] Taylor R, Badcock J, Pargeter K, Lund M, Fred T, Ringrose H, King H, Zimmet P, Bach F, Wang R-L, Sladden T (1992). Dietary intake, exercise, obesity and non-communicable disease in rural and urban populations of three Pacific Island countries. J Am Coll Nutr.

[8] United Nations. Manual X: Indirect Techniques for Demographic Estimation. Department of International Economic and Social Affairs. Population Studies, No. 81.ST/ESA/SER.A/81. United Nations Publication. Sales No. E.83.XIII.2. United Nations, New York, 1983.

[9] Brass W. Demographic data analysis in less developed countries: 1946–1996. Popul Stud 1996;50:451–67. 10.1080/0032472031000149556 11618376

[10] Bradshaw D, Timaes IM, Jamison DT, Feachem RG, Makgoba MW, Baingana FK, Hofman KJ, Rogo KO (2006). Chapter 4: Levels and trends of adult mortality. Disease and mortality in subsaharan Africa.

[11] Rowland DT (2003). Demographic Methods and Concepts.

[12] Coale AJ, Demeny P (1966). Regional Model Life Tables and Stable Population.

[13] United Nations (1981). Model Life Tables for Developing Countries. United Nations Publication, Sales No. E.1981.XIII.7.

[14] Carter K, Cornelius M, Taylor R, Ali S, Rao C, Lopez AD, et al. Mortality trends in Fiji. Aust NZ J Publ Heal. 2011;35(5):412–20. 10.1111/j.1753-6405.2011.00740.x 21973247

[15] Hufanga S, Carter KL, Rao C, Lopez AD, Taylor R (2012). Mortality trends in Tonga: an assessment based on a synthesis of local data. Popul Health Metr.

[16] Carter KL, Hufanga S, Rao C, Akauola S, Lopez AD, Rampitage R, Taylor R (2012). Causes of death in Tonga: quality of certification and implications for statistics. Popul Health Metr.

[17] Carter K, Rao C, Lopez AD, Taylor R (2012). Mortality and cause-of-death reporting and analysis systems in seven Pacific island countries. BMC Public Health.

[18] Kiribati 2000 Census. Table 1–1. Age, Marital Status and Ethnicity by Usual Residence, Kiribati. Available at: http://www.spc.int/nmdi/reports/KIR2000_tbl.pdf. Accessed 20 Feb 2016.

[19] Kiribati 2005 Census. Vol II. Analytic Report. Kiribati National Statistics Office and the Secretariat for the Pacific Community (SPC) Statistics for Development Programme. SPC 2007a.

[20] Kiribati 2010 Census. Volume 2, Analytical report. Kiribati National Statistics Office and the Secretariat for the Pacific Community (SPC) Statistics for Development Programme. SPC 2013. http://www.mfed.gov.ki/statistics/kiribati-document-library. Accessed 20 Feb 2016.

[21] Chiang C (1967). Variance and covariance of life table functions estimated from a sample of deaths. Vital Health Stat 2.

[22] UNICEF 2016. UNICEF Data: Monitoring of the Situation of Children and Women. Child survival. http://data.unicef.org/child-mortality/under-five.html. Accessed 20 Feb 2016.

[23] Boyle P, Parkin DM. Chapter 11. Statistical methods for registries. In: Jensen OM, Parkin DM, MacLennan R, Muir CS, Skeet RG, editors. Cancer Registration: Principles and Methods. Lyon: International Agency for Research on Cancer Scientific Publication No. 95; 1991. https://www.iarc.fr/en/publications/pdfs-online/epi/sp95/sp95-chap11.pdf. Accessed 20 Feb 2016.

[24] Pollard AH, Yusuf F, Pollard GN (1990). Demographic Techniques.

[25] Kiribati MOH PHIN Report. In Annual Report. 2011. http://www.phinnetwork.org/Portals/0/Annual%20Report_Kiribati_2011_Part01.pdf. Accessed 20 Feb 2016.

[26] Coale AJ, Guo G (1989). Revised regional model life tables at very low levels of Mortality. Popul Index.

[27] Murray CJL, Ferguson BD, Lopez AD, Guillot M, Salomon JA, Ahmad O (2003). Modified logit life table system: principles, empirical validation, and application. Pop Stud-J Demog.

[28] MORTPAK. UN Department of economic and Social Affairs. Population Division. The United Nations Software Package for Mortality Measurement. http://www.un.org/en/development/desa/population/publications/mortality/mortpak.shtml. Accessed 20 Feb 2016.

[29] ModMatch. WHO Health statistics and information systems. Software tools. http://www.who.int/healthinfo/global_burden_disease/tools_software/en/. Accessed 20 Feb 2016.

[30] Millennium Development Goals: Kiribati Progress to June 2015. http://www.mfed.gov.ki/sites/default/files/MDG%20Achievements%20June%202015_0.pdf. Accessed 20 Feb 2016.

[31] Timaeus IM (1991). Measurement of adult mortality in less developed countries: a comparative review. Popul Index.

[32] Wang H, Dwyer-Lindgren L, Lofgren KT, Rajaratnam JK, Marcus JR, Levin-Rector A, et al. Age-specific and sex-specific mortality in 187 countries, 1970–2010: a systematic analysis for the Global Burden of Disease Study 2010. Lancet. 2012;380(9859):2071–94. 10.1016/S0140-6736(12)61719-X 23245603

[33] United Nations International Children’s Emergency Fund (UNICEF). Birth Registration in the Pacific, UNICEF Pacific Office, Fiji. 2005. http://www.unicef.org/pacificislands/BR_Report_Part1.pdf. Accessed 20 Feb 2016.

[34] WHO Western Pacific Region Kiribati country profile. 2011. http://www.wpro.who.int/countries/kir/12KIRpro2011_finaldraft.pdf. Accessed 20 Feb 2016.

[35] United Nations Department of Economic and Social Affairs/Population Division. World Population Prospects: The 2015 Revision, Volume II: Demographic Profiles. Kiribati. http://esa.un.org/wpp/Demographic-Profiles/pdfs/296.pdf. Accessed 20 Feb 2016.

[36] National Minimum Development Indicators (NMDI). Vital Statistics Indicators, Public Health NMDIs. Pacific Regional Information System, Statistics for Development, Secretariat of the Pacific Community (SPC). http://www.spc.int/nmdi/vital_statistics. Accessed 20 Feb 2016.

[37] Institute for Health Metrics and Evaluation. GBD Profile: Kiribati. Institute for Health Metrics and Evaluation. GBD 2010. 61 yrs. Seattle. http://www.healthmetricsandevaluation.org/sites/default/files/country-profiles/GBD%20Country%20Report%20-%20Kiribati.pdf. Accessed 20 Feb 2016.

[38] The Brisbane accord group and the Pacific vital statistics action plan (2011–2014). In: Pacific Vital Statistics Action Plan. 2011. http://www.uq.edu.au/hishub/docs/VITAL-STATS-OUTLINE-FINAL.pdf Accessed 20 Feb 2016.

[39] Carter K, Soakai S, Taylor R, Gadabu I, Rao C, Thoma K, et al. Mortality trends and the epidemiological transition in Nauru. Asia Pac J Public Health. 2011;23(1):10–23. 10.1177/1010539510390673 21169596

