# Supplementary material for: Mortality and life expectancy in Kiribati based on analysis of reported deaths
Source: Popul Health Metr. 2016 Feb 29;14:3. doi: 10.1186/s12963-016-0072-6 (PMC4772294; doi:10.1186/s12963-016-0072-6)
Supplement: Additional file 1: — PopLifeTabKir2000-09. (DOCX 52 kb) [file 12963_2016_72_MOESM1_ESM.docx]

**Additional file 1: Populations and life tables, Kiribati 2000-2009**

**Mortality and life expectancy in Kiribati based on analysis of reported deaths**

Karen L Carter (1,4), Tibwataake Baiteke (2), Tiensi Teea (3), Teanibuaka Tabunga (2), Kabuaua Temaava (3), Mantarae Itienang (3), Chalapati Rao (5), Alan D Lopez (6), Richard Taylor (7)

(1) School of Population Health, Faculty of Health Sciences, University of Queensland, St Lucia, Brisbane, Queensland 4072, Australia

(2) Kiribati Ministry of Health (MoH), Tarawa, Kiribati

(3) Kiribati Civil Registration Office (CRO), Ministry of Women, Youth and Communities, Tarawa, Kiribati

(4) Secretariat of the Pacific Community (SPC), B.P. D5 Noumea Cedex , 98848, New Caledonia

(5) Global Health Division, Research School of Population Health, Australian National University (ANU), Acton, Canberra, Australian Capital Territory (ACT) 2601, Australia

(6) Melbourne School of Population and Global Health, University of Melbourne, Parkville, Melbourne, Victoria 3010, Australia

(7) School of Public Health and Community Medicine (SPHCM), University of New South Wales (UNSW), Randwick, Sydney, New South Wales 2052, Australia

**Kiribati census populations and interpolations**

**Kiribati male populations and interpolations 2000-2009**

**Kiribati female populations and interpolations 2000-2009**

Exponential interpolations between censuses

**Kiribati life tables based on reconciled (unique) deaths**

**Kiribati males 2000-2004**

**Kiribati males 2005-2009**

**Kiribati females 2000-2004**

**Kiribati females 2005-2009**

N_x_ Population, d_(adj)_ Reconciled (unique) deaths with unknown age redistributed into age groups
